# Harmonization of multi-site diffusion tensor imaging data for cervical and thoracic spinal cord at 1.5 T and 3 T using longitudinal ComBat

**DOI:** 10.1038/s41598-023-46465-6

**Published:** 2023-11-13

**Authors:** Devon M. Middleton, Yutong Li, Andrew Chen, Russell Shinohara, Joshua Fisher, Laura Krisa, Mark Elliot, Scott H. Faro, John H. Woo, Adam E. Flanders, Feroze B. Mohamed

**Affiliations:** 1 Department of Radiology, Thomas Jefferson University, 909 Walnut Street, First Floor COB, Philadelphia, PA 19107 USA; 2 Sidney Kimmel Medical College, Thomas Jefferson University, Philadelphia, PA USA; 3 Center for Biomedical Image Computing and Analytics (CBICA), University of Pennsylvania, Philadelphia, PA USA; 4 Elsevier, Inc., Philadelphia, PA USA; 5 Department of Occupational Therapy, Thomas Jefferson University, Philadelphia, PA USA; 6 Department of Radiology, University of Pennsylvania, Philadelphia, PA USA

**Keywords:** Diagnostic markers, Magnetic resonance imaging

## Abstract

MRI scanner hardware, field strengths, and sequence parameters are major variables in diffusion studies of the spinal cord. Reliability between scanners is not well known, particularly for the thoracic cord. DTI data was collected for the entire cervical and thoracic spinal cord in thirty healthy adult subjects with different MR vendors and field strengths. DTI metrics were extracted and averaged for all slices within each vertebral level. Metrics were examined for variability and then harmonized using longitudinal ComBat (longComBat). Four scanners were used: Siemens 3 T Prisma, Siemens 1.5 T Avanto, Philips 3 T Ingenia, Philips 1.5 T Achieva. Average full cord diffusion values/standard deviation for all subjects and scanners were FA: 0.63, σ = 0.10, MD: 1.11, σ = 0.12 × 10^−3^ mm^2^/s, AD: 1.98, σ = 0.55 × 10^−3^ mm^2^/s, RD: 0.67, σ = 0.31 × 10^−3^ mm^2^/s. FA metrics averaged for all subjects by level were relatively consistent across scanners, but large variability was found in diffusivity measures. Coefficients of variation were lowest in the cervical region, and relatively lower for FA than diffusivity measures. Harmonized metrics showed greatly improved agreement between scanners. Variability in DTI of the spinal cord arises from scanner hardware differences, pulse sequence differences, physiological motion, and subject compliance. The use of longComBat resulted in large improvement in agreement of all DTI metrics between scanners. This study shows the importance of harmonization of diffusion data in the spinal cord and potential for longitudinal and multisite clinical research and clinical trials.

## Introduction

Diffusion MRI (dMRI), including diffusion tensor imaging (DTI), of the spinal cord is increasingly studied with respect to traumatic and non-traumatic spinal cord injury, degenerative diseases, and other pathologies^1–12^. Numerous reports have been published detailing spinal cord DTI data in adults^2,3^ and pediatric subjects^4–8^. DTI parameters including fractional anisotropy (FA), mean diffusivity (MD), axial diffusivity (AD), and radial diffusivity (RD) have shown to be good indicators of normative white matter microstructure as well as predictors of axonal loss and demyelination in adverse cases such as spinal cord injury in human and animal studies^3,9–12^.

DTI metrics have the potential to serve as biomarkers for injury or disease, but quantitative interpretation of DTI can be complicated, particularly in the case of the spinal cord. In contrast to the brain, the spinal cord experiences greater physiologic motion, and requires high in-plane resolution, resulting in decreased signal-to-noise in magnetic resonance (MR) images. Reduced field-of-view (rFoV) diffusion weighted imaging sequences have demonstrated the ability to reduce distortions and artifacts that afflict the more common Echo Planar Imaging (EPI) sequences^5,13,14^. This rFoV is especially helpful for small structures such as the spinal cord that are susceptible to geometric distortions and is now commonly available across all major MRI vendor platforms. Further complicating matters is the fact that quantitative imaging can be challenging to replicate across different MR scanners due to differences in magnetic field strength, gradient performance, pulse sequence designs, processing techniques and calculation methods. DTI has been studied extensively in the brain and variance between DTI metrics is a complicating factor when comparing data from different trials, sites, and scanners. A further confounding factor is the lack of a gold standard for DTI due to the fact that the metrics obtained are proxy measures for a microstructure which can only be verified by histology or other ex-vivo techniques. Thorough examination of the ex-vivo human and animal^15–20^ spinal cord has been correlated with MR imaging and shown good results, but it remains difficult to determine confidence in comparing in-vivo data acquired with differing environments. Further, if the goal is ultimately to allow DTI to be used in a clinical environment, the limitations of a broad set of MR site capabilities must be considered as stringent research level controls cannot be assured in all clinical settings.

Few studies have been conducted to show spinal cord DTI reproducibility within scanner^4,14^. Additionally, there are a series of multicenter studies that test inter-vendor and inter-field strength effects on DTI of the brain^21–25^. However, to our knowledge no studies have been performed to examine the reproducibility between different scanner manufacturers and different field strengths for the cervical and thoracic spinal cord. In this study, we examined the variability of DTI of the spinal cord between MR scanners of differing field strengths, hardware capabilities, and manufacturers by scanning a sample of thirty healthy adult subjects on four MR scanners at field strengths of 1.5 T and 3 T.

The difficulty in combining DTI data from different studies is an additional challenge, and methods have been developed to harmonize this data between different data sets. The ComBat technique, an empirical Bayesian method, is one such approach^26^. Originally developed for use in genomics, it has been adapted for use in brain DTI with promising results^27^. As a further refinement, Longitudinal ComBat (longComBat) was developed to consider scanner effects and time as variables in harmonization of data^28^. In this study, we also demonstrate the efficacy of longComBat in decreasing scanner effects on the data from different scanners and field strengths. We show that harmonization of the diffusion data in the human spinal cord is a critical step towards opportunities for longitudinal and multisite clinical research and clinical trials.

## Methods

### Subject recruitment

Thirty healthy adult volunteers were recruited for this study. Female subjects (n = 16) ranged in age from 21 to 30, with a mean age of 24.0, and male subjects (n = 14) ranged from 21 to 26 with a mean age of 23.7. Each subject met each of the following inclusion criteria: age between 20 and 30, no history of spinal cord injury, no known neurologic disease such as stroke, traumatic brain injury, multiple sclerosis, neuromyelitis optica, lupus, sarcoidosis, or central nervous system infection, no neurologic complaints or symptoms referable to the spinal cord, and no contraindications for MR imaging including metallic or electronic implants not deemed safe. Subjects completed a written questionnaire indicating any known neurologic conditions, history of back/neck/head injury requiring hospitalization, treatment for muscle weakness, history of loss of sensation, and other medical history in order to assess eligibility. Subjects were recruited using an IRB approved announcement in graduate courses and referrals from study coordinators. All volunteers were educated about the study risks and informed consent was obtained in accordance with IRB approved protocol. This study followed the Declaration of Helsinki and Good Clinical Practice (GCP) standards, and was approved by the institutional review boards of Thomas Jefferson University and the University of Pennsylvania.

### Imaging

Each volunteer was scanned using a total of four scanners; site 1 included a Siemens 3 T Prisma and Siemens 1.5 T Avanto, site 2 included a Philips 3 T Ingenia and a Philips 1.5 T Achieva. The subjects were scanned on both scanners at each site no more than two weeks apart. Scanning at site 2 was performed by a single research assistant trained by senior radiologist and MR physicist investigators, and scanning at site 1 was performed by dedicated research MR technologists under the guidance of the trained research assistant. All scanning protocols included a three-plane localizer, a large field-of-view (FOV) T2 weighted fast spin echo sagittal acquisition, and am axial T2* weighted multi-echo gradient echo (GRE) for purposes of anatomic localization along with the localizer and vertebral level determination. DTI data were collected with axial slices matched to the T2* GRE acquisition. DTI and T2* GRE images were collected by acquiring two overlapping slabs, prescribed for the T2* GRE images in order to cover the cervical C1 to upper thoracic and thoracic down to T12/L1 intervertebral disc level. At least one vertebral level of overlap was present between the two slabs to ensure full coverage.

rFoV based EPI sequences were employed for DTI data collection on scanners capable of these sequences. All scans were performed using vendor provided pulse sequences available of the scanners. Gradient calibration was not performed on scanners prior to imaging. DTI scans were performed using the ZOOM sequence which employs cross sectional RF excitations on the Philips scanners and the ZOOMit technique using spatially selective 2DRF excitations on the Siemens 3 T. No rFoV technique was available on the Siemens 1.5 T scanner but saturation bands were used to suppress signal from the throat region in the full FoV (fFoV). An additional fFoV acquisition was performed on the Philips 1.5 T to examine if better agreement would be found with the fFoV on the Siemens 1.5 T, bringing the total number of DTI acquisitions to 5 per subject. Cardiac gating was used at site 1 for DTI acquisition using a pulse oximeter but was unavailable at site 2 at the time of scanning. The DTI parameters for each scanner are outlined in the following Table 1.

**Table 1:** DTI Acquisition Parameters for all scans.

	Siemens 3 T	Siemens 1.5 T	Philips 3 T	Philips 1.5 T rFOV	Philips 1.5 T fFOV
n directions	20	20	20	20	20
b0 volumes	2	2	2	2	2
b-value (s/mm^2^)	800	800	800	800	800
Voxel size (mm)	1.0 × 1.0 × 6.0	1.5 × 1.5 × 6.0	0.8 × 0.8 × 6.0	1.375 × 1.375 × 6.0	1.42 × 1.42 × 6.0
FoV (mm)	164 × 47	200 × 200	256 × 66	110 × 110	250 × 250
Axial slices	40	40	40	40	40
TR (ms)	7800	5900	7900	6000	6000
TE (ms)	117	85	86	86	120
Acquisition time	8:45	8:43	7:21	7:12	7:12

### Post processing

Following image acquisition, the raw data were corrected for motion that arose from patient movement, cardiac and respiratory movement, as well as eddy current induced distortion. Motion artifacts and eddy current distortions were corrected using FSL’s eddy tool which is part of the FMRIB software library^29^. After correction, a mean-b0 image was generated for use in tensor estimation.

To estimate the tensors, a non-linear implementation of the robust estimation of tensor by outlier rejection (RESTORE) method was used. The RESTORE method attempts to reduce the influence outliers by iteratively re-weighting the signal from diffusion weighted images based on residuals after each round of tensor estimation^30,31^. To measure the DTI metrics, FA, MD, AD, and RD, ROIs were manually drawn at each axial slice on the grayscale FA maps covering the entire cord cross section along the entire cervical and thoracic spinal cord by a research assistant trained by neuroradiologists. Slices exhibiting corruption due to motion artifact or misregistration on visual inspection were not included; i.e. in cases where the spinal cord was not visible or was severely degraded in FA maps. No other omission of slices was performed. Mean ROI values were calculated at each slice and averaged for all slices within the range of a vertebral body as identified by localization with T2 weighted sagittal and T2* weighted axial images.

After processing, tensor estimation, and ROI definition/localization, DTI metrics FA, MD, AD, and RD were compiled for all subjects by scanner/acquisition and vertebral level for further analysis. Average values and standard deviation for all subjects and acquisitions was calculated for whole cord and by vertebral level. Coefficients of variation were calculated as the standard deviation divided by the mean between scanners by level for each subject and averaged for all subjects by vertebral level. Pearson correlations for average values by vertebral level were calculated between scanners using Prism software. Intra-class correlation coefficients (ICCs) were also calculated using ICC(3,1) as defined by Shrout and Fleiss^32^ to examine agreement between scanners by vertebral level using R software.

### Harmonization

Data harmonization was performed using the longComBat technique^28^ with each scan was considered a discrete time point. Age and sex were included as covariates, with any remaining factors grouped as scanner effects. All DTI metrics were organized by subject, scanner, and vertebral level prior to applying longComBat. The harmonization was performed using custom scripts written in Python and R. After harmonization, averaged data for all subjects was compared for pre- and post-harmonization to observe changes in agreement. ICC values were also examined pre- and post-harmonization.

## Results

### Attrition

Twenty of the 30 subjects successfully completed the full protocol on all four scanners. Most commonly, subjects withdrew prior to their scan on the Philips 1.5 T Achieva due to scheduling conflicts. All 30 subjects successfully completed the protocol on the Siemens 3 T Prisma, 29 of 30 for the Siemens 1.5 T Avanto, 28 of 30 on the Philips 3 T Ingenia, and 22 of 30 on the Philips 1.5 T Achieva. In total, out of a planned 150 DTI sequences (5 per subject), 137 were acquired. A total of 125 slices were rejected with 8238 slices used for all acquisitions for an approximate rejection rate of 2%.

### Averaged values

**Figure 1:**
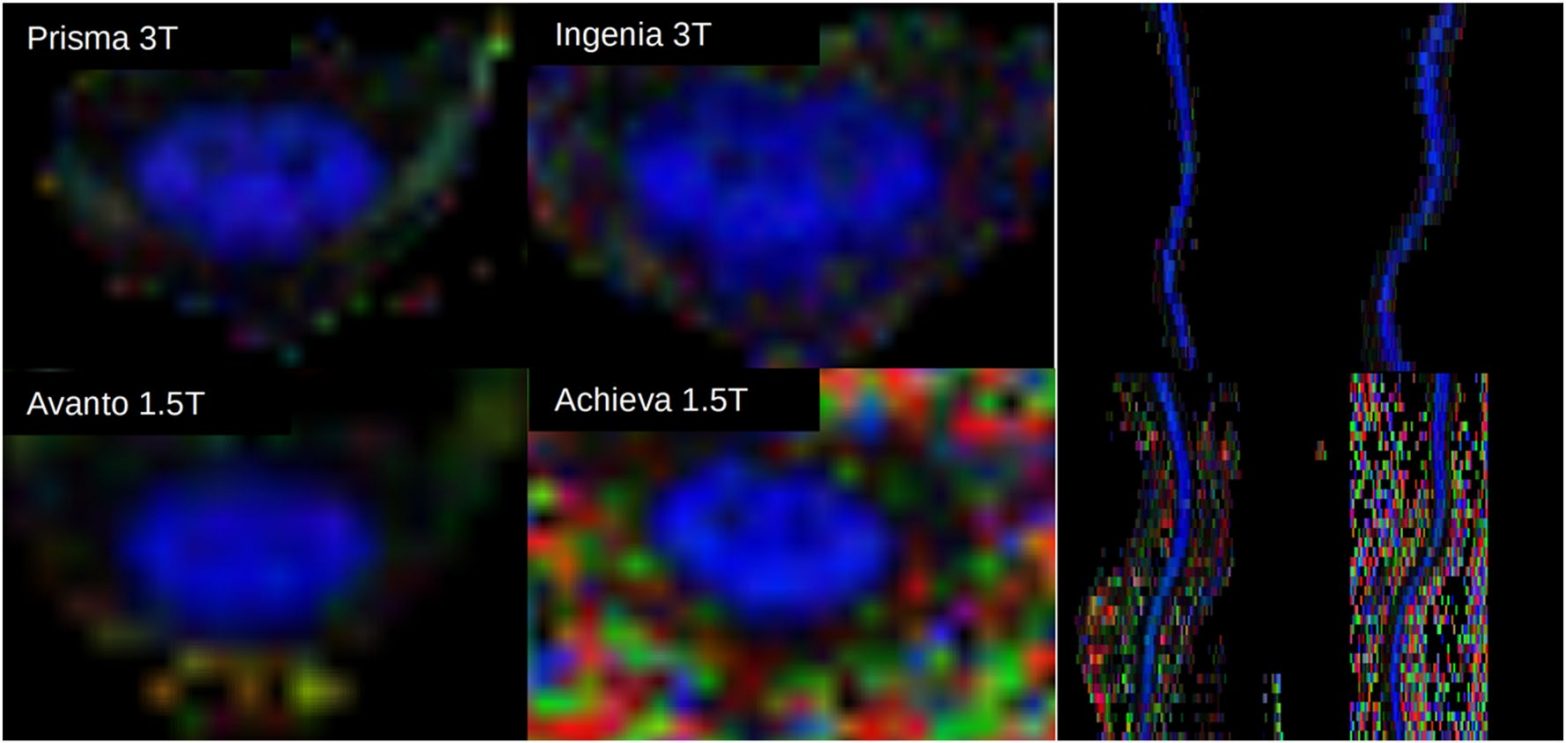
Representative color FA maps at the C3 vertebral level for a single subject from all four scanners (left), as well as sagittal midline reconstructions of the cervical through upper-thoracic spinal cord (right).

**Figure 2:**
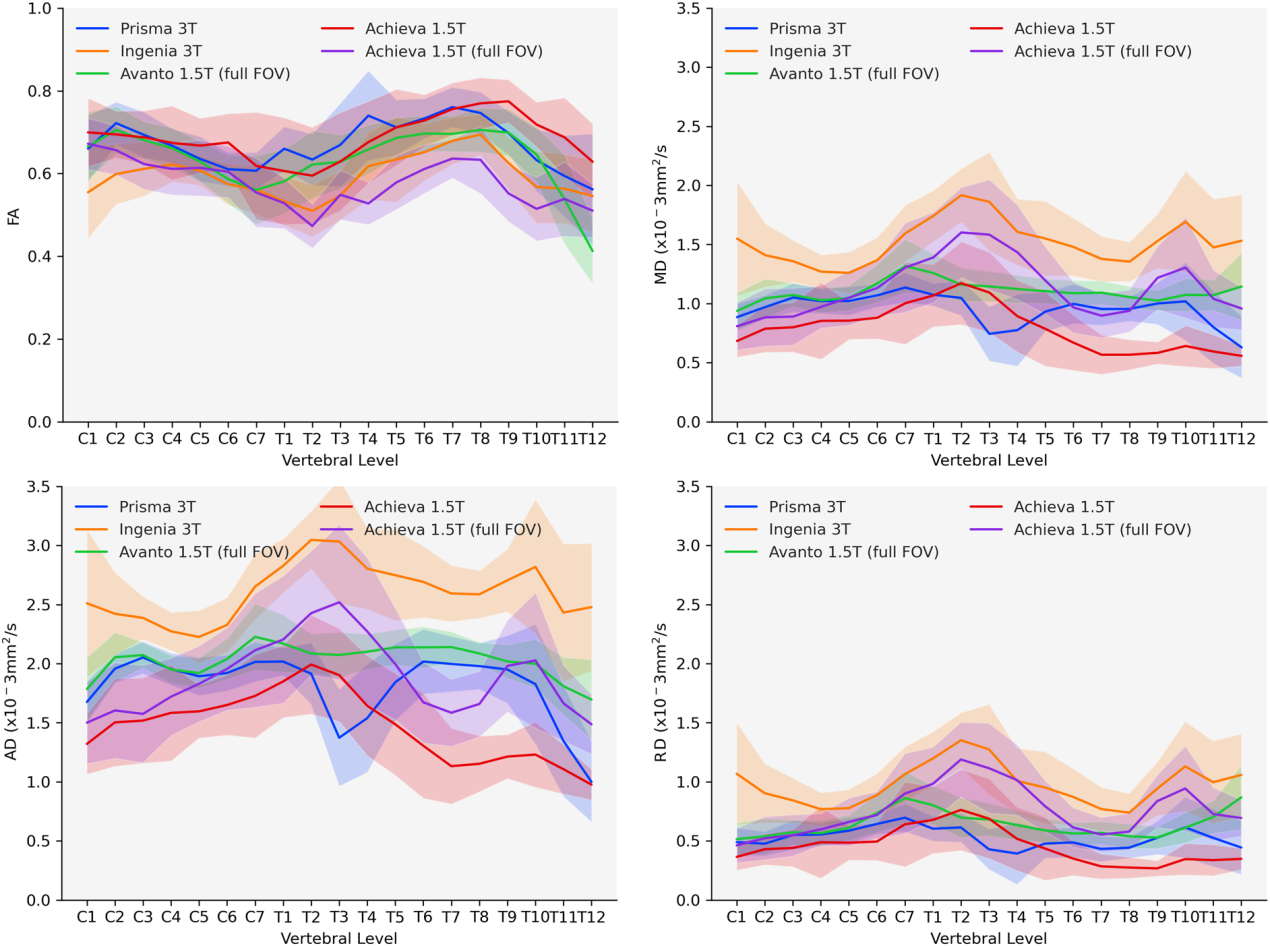
Average FA, MD, AD, and RD for all subjects by scanner/acquisition with standard deviation shown by shaded regions. All sequences are small field of view except where indicated.

FA maps generated showed varying degrees of white/gray matter separation along the length of the cord with higher resolution 3 T scans showing better delineation (Fig. 1). Average full cord values/standard deviation for all subjects and scanners were FA: 0.63, σ = 0.10, MD: 1.11, σ = 0.12, AD: 1.98, σ = 0.55, RD: 0.67, σ = 0.31; diffusivities given as × 10^–3^ mm^2^/s. FA metrics averaged for all subjects by level were relatively consistent across scanners (Fig. 2), but large variability was found in diffusivity measures, particularly in the upper thoracic region where cardiac pulsation can severely complicate imaging. Diffusivity values from the Philips 3 T Ingenia were consistently higher than other scanners.

Despite some consistency in FA absolute agreement between all acquisitions was poor. FA showed moderate to good correlation with r ranging from 0.48 to 0.86. Diffusivity values were considerably more variable in agreement, ranging from 0 to 0.82 depending on metric and scanner comparison. The poorest correlations were generally found in non-gated acquisitions. Coefficient of variation (CoV) in DTI metrics were also examined for scanner averages by vertebral level. In general, FA was again more consistent ranging from 9 to 22%, with diffusivity CoV in the range of 12–48%. ICC values showed moderate agreement in FA (ICC = 0.63), but generally poor agreement in diffusivity values (0.37 < ICC < 0.48). Despite the moderate to poor agreement in the original data, the harmonized data showed large improvements.

### Harmonization results

**Figure 3:**
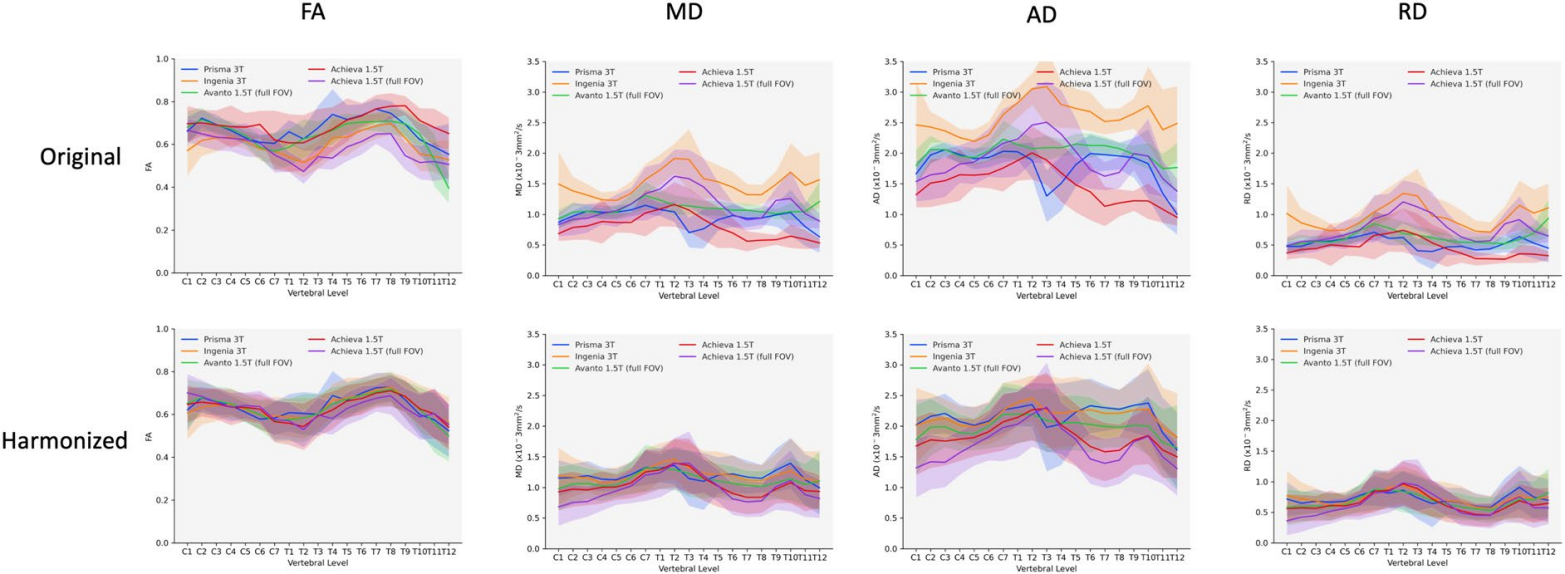
Average FA, MD, AD, and RD for all subjects by scanner/acquisition with standard deviation shown by shaded regions for original vs. harmonized data. All sequences are small field of view except where indicated.

**Figure 4:**
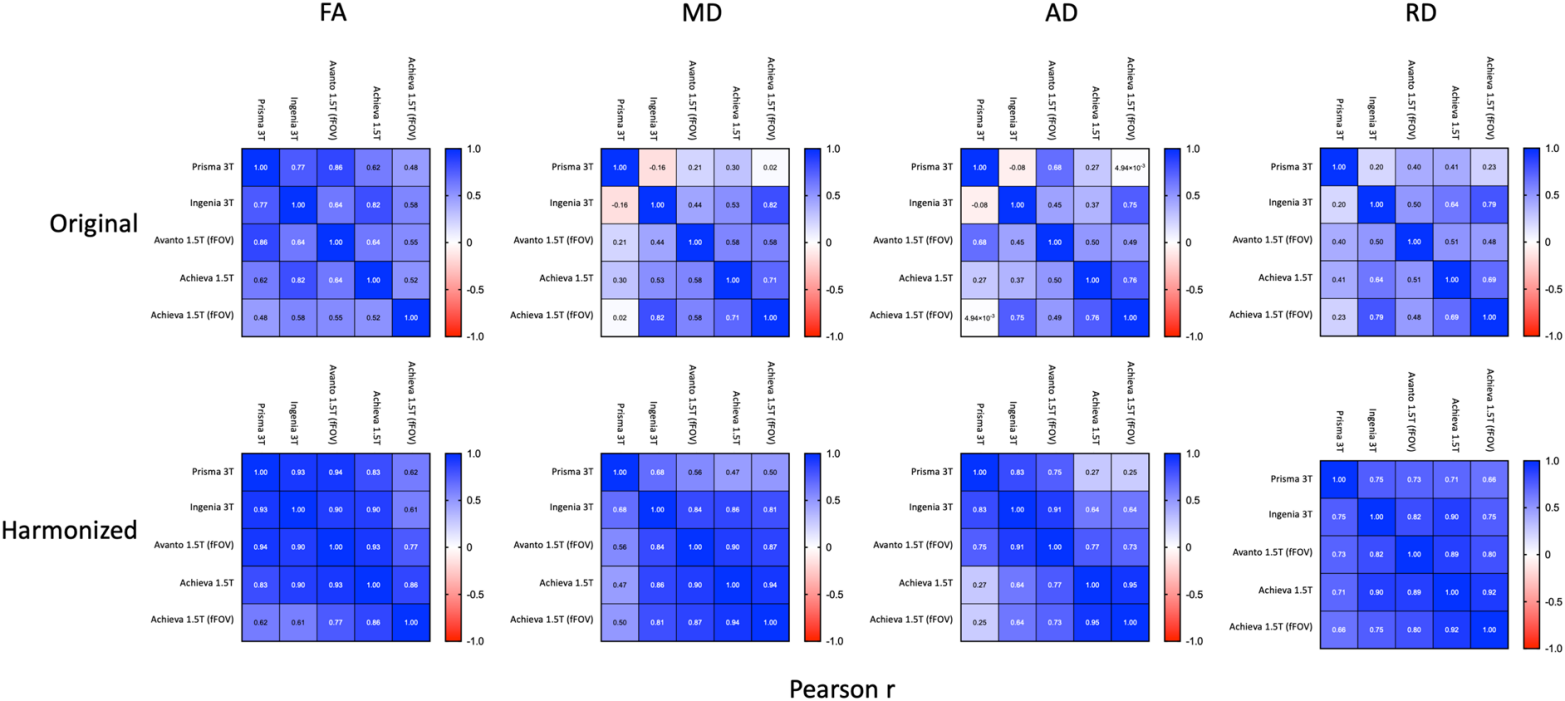
Pearson correlations between all scanners based on average value by vertebral level showing original and harmonized results.

**Figure 5:**
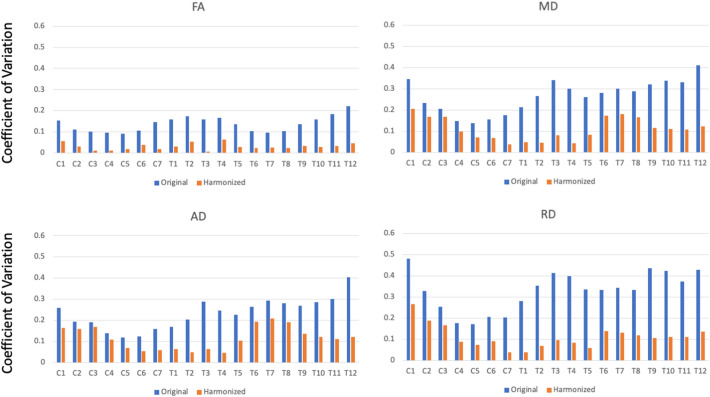
Coefficient of average scanner values for all DTI metrics by vertebral level.

Harmonized data showed marked improvements in agreement between scanners. In general, average values were more similar for all DTI metrics (Fig. 3) along the length of the SC. Inter-scanner correlations of averaged DTI metrics improved drastically, most notably in diffusivity measures (Fig. 4). Of particular note was the improvement in the upper thoracic region where different scanners exhibited the most pronounced variation in DTI metrics. CoV values for all metrics were also substantially improved post-harmonization, with FA in particular showing very low values across scanners (Fig. 5).

ICC values also showed considerable improvement with good agreement in FA, and moderate to good agreement in diffusivity measures (Table 2).

**Table 2:** ICC values at 95% confidence interval for all acquisitions, showing lower (LB) and upper (UB) bounds.

	Original			Harmonized		
	LB	ICC	UB	LB	ICC	UB
FA	0.47	0.65	0.82	0.7	0.83	0.92
MD	0.18	0.39	0.63	0.48	0.67	0.83
AD	0.18	0.38	0.62	0.40	0.60	0.78
RD	0.26	0.47	0.70	0.57	0.74	0.87

Values were computed for all metrics by vertebral level.

## Discussion

The harmonization approach using longComBat was effective in improving agreement and reducing variance between datasets acquired from different scanners in this study. Average values were much more consistent post-harmonization, correlations were improved, variance was decreased, and agreement improved substantially as measured by ICC. LongComBat was originally presented in the context of longitudinal study designs, but is a more generally applicable tool. Similar to a longitudinal model based on a mixed effect regression reducing to a simple repeated measures model when no time effect is included, longComBat without a time effect has been validated for dMRI data of the brain^33^. It has additionally been recommended for use in travelling subject studies^34^.

Multiple issues complicate acquisition of spinal cord DTI data including scanner hardware limitations, small cord size, and critically physiologic motion and subject compliance. An immediately noticeable consequence of physiologic motion is the spiking in the upper thoracic region in non-gated acquisitions. In the absence of cardiac gating, this is not unexpected as motion artifact and misalignment can result in artifactually exaggerated attenuation in diffusion weighted images thus creating spikes in the region most impacted by cardiac motion. Interestingly, gating seems to have a potentially opposite effect in the upper-thoracic region with a pronounced decrease in the Siemens 3 T Prisma, and slight downward trend for the Siemens 1.5 T Avanto.

Diffusivities on average exhibited considerable variability between scanners. This is expected in some respects due to the fact that diffusivity metrics are directly related to absolute attenuation between unweighted and diffusion weighted images. Differences in acquisition parameters will necessarily have some impact on this attenuation along with SNR.

FA values on average for all scanners was more consistent than diffusivities with lower CoV and similar trends for all acquisition averages. Because FA is a normalized metric, it is more resistant to variation in absolute attenuation in diffusion weighted images, provided the variation propagates similarly to all diffusion weighted images for a given acquisition. Considerable variance present in the thoracic and lumbar cord suggests that any inter-scanner variability may also be further confounded by variability in physiologic noise which is not completely resolvable due to SNR dropout. The cervical cord is also a better subject due to the reduced physiologic noise as compared with the thoracic region.

Agreement between the fFoV acquisitions on the two 1.5 T scanners was not necessarily better as compared with rFoV to fFoV as shown in the correlation matrix. It is likely that other scanner effects have a more dominant role as compared to the fFoV vs rFoV difference which has shown to be a source of variability in other studies^14,35^.

The impact of cardiac gating is an important consideration in these results. All-subject average data showed an possible overestimation of diffusivity in the upper thoracic region in the absence of gating, but a potential underestimation when it is applied. However, the employment of longComBat was effective in mitigating these effects, with the disagreement resolving considerably. It should be noted that manufacturer and site differed for gated and non-gated acquisitions which may contribute some degree to these differences, and longComBat’s agnostic view of the multiple confounds present may be advantageous in this situation.

While to our knowledge no multi-site trials of DTI of the cervical and thoracic spinal cord have been performed using a travelling cohort, multi-site studies of brain DTI have shown a range of agreement and consistency, generally moderate to good, depending on anatomical region, DTI metric, and variability in scanner. This study contained several significant challenges, including variation in hardware, sequence parameters, and subjects. Differences in achievable sequence parameters, field strength, and gating capabilities all impact the SNR and absolute diffusion attenuation in a DTI acquisition, thus the relatively lower consistency in the original data is not a total surprise. While every effort was made to instruct subjects in remaining still, subject motion was present in a number of scans which confounded the unavoidable problem of physiologic motion from cardiac and respiratory cycles. A pure slice-wise correction scheme is frequently used in spinal cord DTI, and 3D corrections have been shown to be useful in the motion-prone pediatric population^36^. Compensation for this was attempted through the use of a 3D motion correction technique with slice-to-volume correction^29^ and from inspection performed well. The use of different field strengths and sequence parameters is a limitation of the study in terms of strict reliability. However, these differences are also representative of issues present in many multi-center trials in clinical trial and research settings and the improvement in agreement is encouraging.

While a travelling subject scenario is not generally common in multi-center clinical trials, there are situations, especially in spinal cord injury research, where patients are treated and followed up at different institutions after the initial imaging or use different MRI scanners due to challenges in mobility and transportation. In such situations, these results can provide increased confidence that DTI metrics obtained at various scanners or at various institutions can be reliably compared and used for follow-up treatment after application of harmonization. The longComBat method requires a minimum of two scans per scanner, but it does not require overlapping scans of the same subjects on different scanners or matched number of total subjects per scanner. The longComBat method was used in this work as it was the most appropriate technique for the cohort available, and because it is most applicable to studies with repeated measures which are greatly needed in human spinal cord injury. Future work on comparisons of long ComBat with cross-sectional harmonization methods for cases of single measurement multisite studies is additionally interesting and warranted.

As spinal cord DTI becomes more broadly examined, site specific limitations in hardware and sequence parameters may prevent reproduction of protocols, particularly in clinical settings. Important work has been performed on the standardization of SC dMRI in attempts to create more consistent scanning protocols and procedures^37^, but in many cases technical limitations or time constraints may make this difficult. In the absence of the ability to thoroughly standardize SC dMRI protocols across sites or datasets, longComBat shows promise as a means of allowing meaningful combination of dMRI data in the SC. If protocols are better standardized, it may remain useful in mitigation of unavoidable scanner effects.

## Data Availability

The data used in this study is available upon reasonable request to the corresponding author.
